# Supplementary material for: Prepollination barriers prevent gene flow between co-occurring bat-pollinated bromeliads in a montane forest
Source: PeerJ. 2025 Aug 22;13:e19652. doi: 10.7717/peerj.19652 (PMC12377363; doi:10.7717/peerj.19652)
Supplement: Supplemental Information 2 — Data are values of the reproductive isolation index (RIF) calculated over four reproductive seasons (see methods for calculations). The studied species were W. ampla (Wa), W. nephrolepis (Wn), W. pedicellata (Wp), and W. subsecunda (Ws). [file peerj-13-19652-s002.docx]

**TABLE S1. E**stimations of the strength of temporal reproductive isolation by phenology among species-pairs from four *Werauhia* species (Bromeliaceae) in a montane forest, Costa Rica. Data are values of the reproductive isolation index (RI_F_) calculated over four reproductive seasons (see methods for calculations). The studied species were *W. ampla* (Wa), *W. nephrolepis* (Wn), *W. pedicellata* (Wp), and *W. subsecunda* (Ws).

|  | **Reproductive season** | | | | **Interannual Average RI_F_** |
| --- | --- | --- | --- | --- | --- |
| **Species pairs** | **2012-2013** | **2014-2015** | **2018-2019** | **2020-2021** |  |
| Wa x Ws | 0.14 | 0.18 | 0.05 | 0.13 | 0.13 |
| Ws x Wa | 0.40 | 0.36 | 0.11 | 0.16 | 0.26 |
| Wp x Ws | 0.10 | 0.37 | 0.61 | 0.30 | 0.35 |
| Wp x Wa | 0.53 | 0.55 | 0.50 | 0.28 | 0.47 |
| Wa x Wp | 0.81 | 0.38 | 0.33 | 0.47 | 0.50 |
| Ws x Wp | 0.62 | 0.43 | 0.62 | 0.54 | 0.55 |
| Ws x Wn | 1.00 | 0.98 | 1.00 | 0.96 | 0.98 |
| Wn x Ws | 1.00 | 0.98 | 1.00 | 0.89 | 0.97 |
| Wn x Wa | 1.00 | 1.00 | 1.00 | 0.87 | 0.97 |
| Wp x Wn | 0.95 | 1.00 | 1.00 | 0.97 | 0.98 |
| Wa x Wn | 1.00 | 1.00 | 1.00 | 0.94 | 0.98 |
| Wn x Wp | 1.00 | 1.00 | 1.00 | 0.97 | 0.99 |
| **Average RI_F_** | **0.712** | **0.686** | **0.684** | **0.624** | **0.677** |
