# Supplementary material for: Prepollination barriers prevent gene flow between co-occurring bat-pollinated bromeliads in a montane forest
Source: PeerJ. 2025 Aug 22;13:e19652. doi: 10.7717/peerj.19652 (PMC12377363; doi:10.7717/peerj.19652)
Supplement: Supplemental Information 3 [file peerj-13-19652-s003.docx]

**TABLE S2.** Contribution to the first four dimensions of four morphological traits associated to flower size variable after a principal components (PCA) from the bromeliad species: *Werauhia ampla, W. nephrolepis, W. pedicellata and W.*  *subsecunda* in a montane forest, Cerros de la Carpintera, Costa Rica.

| **Variable** | **PC1** | **PC2** | **PC3** | **PC4** |
| --- | --- | --- | --- | --- |
| Corolla length | 25.9 | 9.3 | 64.2 | 0.5 |
| Corolla mouth | 21.7 | 78.1 | 0.2 | 0.0 |
| Stamen length | 26.3 | 5.6 | 12.0 | 56.1 |
| Pistil length | 26.2 | 6.9 | 23.6 | 42.3 |
